# The health service use of aged rural-to-urban migrant workers in different types of cities in China

**DOI:** 10.1186/s12913-021-06638-3

**Published:** 2021-06-28

**Authors:** Yating Xie, Qiuju Guo, Yang Meng

**Affiliations:** 1 Research Center for Social Governance Innovation, Henan Agricultural University, Zhengzhou, 450006 Henan China; 2 Elder Service Research Center, School of Sociology, Huazhong University of Science and Technology, Luoyu Road #1037, Hubei 430074 Wuhan, China; 3 The Institute for Population and Development Studies, Xi’an Jiaotong University, 710049 Xi’an, Shaanxi China

**Keywords:** Migration destination cities, Health service use, Aged rural-to-urban migrant workers, Healthcare inequality

## Abstract

**Background:**

The association between different types of cities and the use of health services by aged migrant workers in China has not been widely reported in previous studies. This article aims to focus on rural-to-urban migrant workers in China aged 50 years and older to examine the relationship between the region of these migrant workers’ destination city (eastern, central or western) and migration city type (first-tier, second-tier, third-tier and smaller cities) and their use of health services (e.g., establishing health records, participating in health education, and seeking medical treatment when ill).

**Methods:**

This study’s data were obtained from China Migrants Dynamic Survey in 2017. A total of 14,732 rural-to-urban migrant workers aged 50 years and older were included in the analysis; 6,938 of the migrant workers were either ill or had recently experienced physical discomfort. A chi-square test and binary logistic regression were performed to explore the associations between these rural–urban migrants’ destination cities and their use of health services.

**Results:**

This study found that aged rural migrant workers who moved to the east or to first- or second-tier cities were less likely to establish health records, participate in health education programme, and seek medical care.

**Conclusions:**

Migrant destination cities are linked to the use of local health services by migrant workers aged 50 years and older in China. We found that aged migrant workers who migrated to relatively developed regions and cities accessed fewer health services. Such results signify that more attention should be paid to aged migrant workers’ use of health services in economically developed regions and cities, to eliminate regional differences in healthcare inequality.

## Background

Under the trend of long-term population mobility from rural to urban areas in China, the number of migrant workers aged 50 years or older has been increasing and accounted for 22.4% of China’s 288 million migrant workers in 2018 [1]. Unlike middle-aged and young migrant workers, aged migrant workers are more likely to be engaged in lower paid, physically demanding, and high-risk labour industries [2] that increase their health risks. Therefore, it is necessary to pay attention to the health service use among migrant workers aged 50 and older and the factors that influence their use of health services. Previous studies have already examined migrants’ use health services in China in terms of health records, health education, and medical service use [3–5]. Most of these studies have focused on the micro factors that closely relate to people at an individual level, like age, gender, socioeconomic status, and marital status [4–6], but these studies have not paid enough attention to macro-regional factors, such as regions of destination city or city types, which may be more relevant for the formulation of applicable or region-specific social policies.

Previous research has already established that place of residence, especially whether people live in rural or non-rural areas, is associated with disparities in health services [7–11]; further, similar studies in China have mostly analysed healthcare inequality from the perspective of *hukou* (household registration) [3–5]. Earlier studies have determined that most migrant workers have difficulty obtaining the same health services as locals in their destination cities; however, little is known about health service use in different migration destination cities by aged migrants from rural areas. Research on the use of health service employing a macro regional living analytical perspective can provide evidence on the relationship between city type and the health service use in the context of population movement. This will help in identifying whether there are health inequalities across cities, as well as the effort needed to improve the use of public health services among the aged rural-to-urban migrant workers in China and by extension other developing countries.

The present study aims to focus on rural-to-urban migrant workers aged 50 years and older in China and examines the relationship between the geographical region of these workers’ migration destination city (eastern, central, or western), city type of destination city (first-tier, second-tier, third-tier, and smaller cities) and their use of health services, including establishing health records, participating in health education, and seeking medical treatment when ill.

## Literature review

### Residence place and health services

Previous studies that mainly focused on place of residence in rural and urban areas found it to be a vital determinant of the use of public health services [9–13]. Many earlier studies have found that rural dwellers encounter more barriers to accessing health services than urban dwellers, such as long travelling distances and higher costs of healthcare services [10, 11, 14]. Inequality in access to public health services also exists in China; when rural dwellers try to access healthcare services, they tend to encounter more financial and physical barriers, and they experience less access than urban dwellers [15, 16]. In addition to these obstacles, rural and urban disparities in health service systems are a critical reason underlying the unequal use of health services between urban and rural areas.

China has a longstanding dual urban–rural structure under the household registration system. For example, the social medical security system is based on household registration and employment, that is, only those with an urban household registration or formal urban employment can access the urban medical insurance [6, 16]; the majority rural residents can only participate in the new rural cooperative medical scheme (NCMS) [16]. The NCMS has more limited financing compared to urban social medical insurance, reflecting large deductibles, low ceilings, and high coinsurance rates [17]. Consequently, rural residents and most migrant workers find it difficult to access the same health services as urban residents [3, 4]. Most of rural-to-urban migrant workers who engage in informal employment are excluded from the existing medical security system in urban areas [18, 19]. Existing studies have found that when compared with urban residents, migrant workers can only access limited health service programmes due to household registration restrictions, low income, and a lack of local medical insurance [20, 21].

Previous research has determined that place of residence is associated with health services use, but most of these studies only analysed differences between rural and urban areas, without clarifying differences between cities. Moreover, due to the restrictions of *hukou* in China, most previous studies have analysed migrant workers’ use of health services from the perspective of *hukou* rather than from the perspective of actual place of residence. Removing the interference of *hukou* and analysing aged migrant workers’ use of health services in different cities will help further examine the relationship between place of residence and use of healthcare services.

### Migrant workers’ use of health service destination cities in China

To reduce inequality in the use of health services, the Chinese government has amended certain public health services and social security systems by including the characteristics of China’s floating population. The National Health Commission of China has launched and promoted equal access to basic public health and family planning services for cities’ floating population by establishing health records within the relevant jurisdiction, conducting health education programmes, strengthening the prevention and control of infectious diseases, and establishing health management records for children and pregnant women. At present, 20% of China’s floating population of migrant workers have established health records in their current place of residence [6].

Meanwhile, government departments have been encouraging migrant workers to access urban workers’ medical insurance and the new rural medical insurance (NCMS). According to the National Development and Reform Commission, the coverage for basic medical insurance in China reached 95% in 2011 [22]; further, 17% of migrant workers had acquired urban medical insurance by the end of 2016 [23]. Existing studies have posited that implementing and promoting these medical insurance measures have improved the health of floating populations to a certain extent, and that floating populations who received timely health education and medical treatment have presented better health-related quality of life [24].

Although health services for migrant workers in cities have improved, different destination cities do not only signify different levels of economic development, they also represent different public service supply capacities that could be linked to different levels of the floating populations’ use of health services. The common classification of city types in China is based on economic development: the destination cities of migrant workers are divided into eastern, central, and western regions, or into first-tier, second-tier and third-tier cities. The eastern region generally includes coastal zone areas, such as Jiangsu, Zhejiang, Fujian, and Guangdong; the central region includes Jilin, Hubei, Hunan, and Jiangxi; and the western region includes Sichuan, Yunnan, Guizhou, and Xinjiang. First-tier cities belong to the eastern region and include Beijing, Guangzhou, Shenzhen, Shanghai, and Tianjin, all of which are mega cities with a population of over 10 million people. Second-tier cities mainly include provincial capitals and coastal cities, such as Hangzhou, Nanjing, Qingdao, and Chengdu. Third-tier cities are relatively developed small- and medium-sized cities, such as Lanzhou, Jinhua, and Yangzhou. In terms of natural and economic conditions, the eastern region and first-tier cities are considered to have the best conditions, followed by the central region and second-tier cities, and finally by the western region and third-tier cities. Cities with different degrees of development inevitably lead to differences in public health services.

A certain relationship exists between different types of destination cities and health services use, which might be due to the following reasons. First, concerning the inflow of resources and service capacity in the destination region, previous studies have revealed that the eastern regions have the strongest public service capacities (including for healthcare services), followed by the central and western regions [3]. First- and second-tier cities are mainly located in the eastern region, and basic public service levels in first- and second-tier cities is significantly higher than in small- and medium-sized cities [3]. Differentiated public service capability might lead to differentiated health service use among older migrant workers. Second, concerning policy implementation, the healthcare service centre of a local urban community generally undertakes the equalisation of basic public health and family planning services. Most of the floating populations are concentrated in the eastern region and first-tier cities. The size and mobility of floating populations may increase the difficulty of equalising public health services, thus affecting older migrant workers’ use of health services. Studies have indicated that residents in smaller cities use public services more frequently with a higher level of satisfaction in public services [25]. Conversely, floating populations in larger cities are known to receive fewer public services [26]. Further, some health services have geographical limitations. For example, regarding medical treatment services, medical insurance tends to have tighter restrictions for different geographical and medical institution levels. The reimbursement rate is generally higher for local grassroots and low-level medical and health institutions, while it is lower for medical treatment in high-level medical institutions and across provinces; further, trans-provincial medical care reimbursement are subject to strict procedural regulations [27]. Therefore, migration regions and cities may influence the medical treatment-seeking behaviours of floating populations. Finally, from the perspective of the migrant workers themselves, the cost of living in different regions and cities also influences their use of health services. Migrant workers in the large cities such as Beijing, Shanghai, and Guangzhou generally choose not to seek medical treatment or self-treatment when they are sick due to the high medical costs [28]. Meanwhile, household registration restrictions and the high houses price in the eastern region and first-tier cities have led to the residential segregation of migrant workers; many migrant workers tend to live in urban villages or suburbs [29, 30]. This residential segregation could affect their use of health services. Previous studies have found that floating populations that live in urban villages and suburbs are less likely to benefit from public health services than those living in urban areas and communities with neighbourhoods dominated by local residents [31, 32]. These findings indicate a certain relationship between types of destination cities (including migration regions and city types) and aged migrant workers’ use of health services.

To verify this relationship, this article uses survey data from a nationwide floating population to test the relationship between the migration region of aged migrant workers (eastern, central, or western), the migration city types of aged migrant workers (first-tier, second-tier, third-tier and smaller cities) and the use of health services (e.g., establishing health records, participating in health education, and seeking medical treatment). This exploration offers a theoretical and practical basis for promoting a service equalisation policy and for reducing the health inequality in China’s floating populations.

### Data and methodology

The data for this study were obtained from the 2017 China Migrants Dynamic Survey, conducted by the National Health and Family Planning Commission. The participants of the survey were floating populations aged 15 years and older who had been living in a destination area for more than one month. The survey included the collection of basic information, such as information related to family members, migration trends, willingness to settle permanently, employment characteristics, use of basic public health services, and marital and childbirth status. The survey adopted a stratified, multistage, probability proportional to size (PPS) sampling approach, covering 31 provinces. Counties were selected in the first stage, towns or streets were extracted in the second stage, communities in the third stage, and individuals in the fourth stage. The survey was based on one-on-one interviews with professionally trained staff, with a total sample size of 169,000. To analyse aged migrant workers’ use of health services specifically, only rural-to-urban floating populations aged 50 years and older were retained for this study. After deleting the irrelevant population samples from the data, the final sample size used in this study was 14,732. Among these, the sample of people who sought medical treatment when becoming sick included those who had recently been sick or unwell (with a sample size of 6,938).

### Measures

#### Dependent variables

In this study, use of public health services was the dependent variable and included three types of dichotomous variables: whether to establish health records locally, whether to receive local health education, and whether to seek timely medical treatment in case of illness. The first two actions were performed by local community healthcare centres and not only required the initiative of a policy implementation but also the floating populations’ cooperation, but could also reflect on the supply of health services at a community level. Regarding the third action (seeking timely medical treatment in case of illness), people who went to community public health stations, individual clinics, or hospitals were regarded as accessing timely medical treatment, while people who received no medical treatment or who self-treated without visiting a physician were regarded as not accessing timely medical treatment. Seeking timely medical treatment in case of illness directly reflects a floating population’s use of health services. Further, it can also reveal whether ‘remote medical insurance (migrant workers who seek medical treatment outside their medical insurance areas)’ creates certain obstacles to floating populations’ medical treatment, which ultimately indicates the medical health system’s rationale.

#### Independent variables

Based on the classification determined by the National Bureau of Statistics of China, the independent variables in this study included two types of destination categories: migration regions and migration cities. Regarding migration regions, China was divided into eastern, central, and western regions according to the region’s level of economic development level and the rate of economic development of each province, with the western region as the reference item. Regarding migration cities, the cities were divided into first-tier, second-tier, and third-tier cities and smaller cities, based on the cities’ economic strength, political status, urban population size, and regional influence; third-tier cities and smaller cities were used as the reference item.

#### Control variables

After examining previous research, the present study added additional confounding variables in the Andersen health service utilisation model, which is a well-validated theoretical framework to understand determinants of health services utilisation [33]. The model included three main influencing factors: predisposing, enabling, and needs factors. Predisposing factors included socio-demographic characteristics such as age (treated as a continuous variable), gender (male = 0 and female = 1), education level (illiterate or primary school level = 0, middle school level = 1 and high school level or above = 2); and marital status (no spouse = 0 and married = 1). Enabling factors included migration range (across counties but within a city = 0, across cities but within a province = 1, and across provinces = 2) and whether people had medical insurance and a social security card (no = 0 and yes = 1). Needs factors included the floating populations’ self-assessment of health (unwell = 0, fair = 1, and healthy = 2) and whether people had hypertension or diabetes (no = 0 and yes = 1). The social security card mentioned as one of the enabling factors refers to the card promoted nationwide in China that people use to settle their medical insurance personal accounts, handle endowment insurance, handle unemployment registration, apply for employment training, and perform various other social security actions [34].

#### Analytical strategy

The analysis included two main strategies. First, the descriptive statistics of the independent and control variables were obtained (percentage distributions, means, or standard deviations), and the differences in health service use and control variables of aged migrant workers in different migration regions and types of cities were compared using the χ^2^ test and ANOVA analysis. Second, three separate logistic regressions were used to analyse the relationship between the migration regions and city types and the health services use of migrant workers aged 50 years and older, and all the control variables were adjusted for in the logistic regression models. People with poor health are more likely to use health services; therefore, to exclude the interference of health factors, logistic regression was also used to analyse the relationship between migration destination cities and use of health services among the samples of people whose self-reported health was ‘healthy’ and who did not have hypertension or diabetes.

## Results

### Descriptive analyses

As can be observed in Table 1, many aged migrant workers lived in the western region of China (37.20%) and in third-tier and smaller cities (62.72%). The average age of the sample was 56.16 years, and the main education level was middle school or lower. The proportion of aged male migrant workers was higher than aged female migrant workers. Most of the aged migrant workers were married, had medical insurance, and were in good health. Further, the proportion suffering from hypertension or diabetes was relatively low. This indicates that good health is the premise of mobility.

**Table 1:** Sample characteristics (*n* = 14,372)

Variables	% or Mean (SD)	Variables	% or Mean (SD)
Migration region		Migration range	
Western	37.20	Across counties, within a city	24.33
Central	29.12	Across cities, within a province	30.53
Eastern	33.68	Across provinces	45.15
Migration city type		Medical insurance	
Third-tier and smaller cities	62.72	No	7.24
Second-tier cities	26.87	Yes	92.76
First-tier cities	10.41	Social security card	
Age	56.16 (6.43)	No	58.82
Gender		Yes	41.18
Male	60.09	Self-health assessment	
Female	39.91	Unwell	11.97
Education level		Fair	29.12
Illiterate or primary school	47.63	Healthy	58.91
Middle school	40.74	Hypertension or diabetes	
High school and above	11.63	No	78.94
Marital status		Yes	21.06
No spouse	9.09		
Married	90.91		

Table 2 outlines and compares the differences in health service use among aged migrant workers who migrated to different regions and different types of cities according to the chi-square test results. Of the migrant workers aged 50 years and older, 27.63% had established their health records in the health service centres of their local cities, more than 60% had participated in at least one health education programme, and nearly half of the aged migrant workers had indicated that they would seek medical care in the case of illness. The chi-square test results revealed that migrant workers who moved to economically better regions (e.g., eastern regions and first-tier cities), were less likely to use health services, and the difference was significant.

**Table 2:** Health service use among rural–urban migrants aged 50 years and older in different migration destinations

Variables	Health records (*n* = 14372)		Health education (*n* = 14372)		Medical treatment (*n* = 6938)	
	Yes	No	Yes	No	Yes	No
Average	27.63	72.37	63.30	36.70	52.59	47.41
Migration region						
Western	29.14	70.86	72.10	27.90	55.48	44.52
Central	37.32	62.68	64.32	35.68	53.44	46.56
Eastern	17.58	82.42	52.71	47.29	49.04	50.96
χ^2^ test	***		***		***	
Migration city type						
Third-tier and smaller cities	30.99	69.01	65.89	34.11	55.82	44.18
Second-tier cities	25.89	74.11	61.39	38.61	48.43	51.57
First-tier cities	11.90	88.10	52.67	47.33	44.67	55.33
χ^2^ test	***		***		***	

Note: *** *p* < 0.001

### Multivariable linear regression analysis

Table 3 presents the associations between the migration region and health service use in migration cities, as estimated by the logistic regression analysis. Compared with the western region, aged migrant workers in the central region were more likely to establish health records; however, aged migrant workers in the eastern region were less likely to establish health records. In terms of participating in health education programmes, aged migrant workers in the central and eastern regions were less likely to participate than those in the western regions. In the case of illness, aged migrant workers who moved to the eastern region were also less likely to seek timely medical treatment than those who moved to the western region.

**Table 3:** Association between migration region and health services use, based on a logistic regression analysis

Variables	Health records	Health education	Medical treatment
***Independent variables***			
Migration region (western)			
Central	0.39(0.05)***	-0.35(0.05)***	-0.13(0.06)*
Eastern	-0.64 (0.05)***	-0.90(0.04)***	-0.19(0.06)**
***Control variables***			
* Predisposing*			
Age	0.01(0.00)*	-0.02(0.00)***	-0.00(0.00)
Gender (male)			
Female	0.06(0.04)	-0.12(0.04)***	-0.00(0.05)
Education (illiterate or primary school)			
Middle school	0.06(0.04)	0.25(0.04)***	-0.08(0.05)
High school and above	-0.03(0.06)	0.32(0.07)***	-0.05(0.08)
Marital status (no spouse)			
Married	0.20(0.07)**	0.08(0.06)	0.039(0.09)
*Enabling*			
Migration range (across counties, within a city)			
Across cities, within a province	0.00(0.05)	-0.06(0.05)	-0.26(0.07)***
Across province	-0.03(0.06)	-0.02(0.05)	-0.17(0.07)*
Medical insurance (no)			
Yes	0.23(0.08)**	0.19(0.07)**	0.12(0.10)
Social security card (no)			
Yes	0.52(0.04)***	0.33(0.04)***	0.18(0.05)***
*Needs*			
Self-health assessment (unwell)			
Fair	0.14(0.07)*	0.25(0.06)***	-0.37(0.07)***
Healthy	0.22(0.07)***	0.33(0.06)***	-0.65(0.07)***
Hypertension or diabetes (no)			
Yes	0.24(0.05)***	0.10(0.05)*	0.24(0.06)***
Constants	-2.37(0.28)***	1.44(0.22)***	0.61(0.30)*
Log likelihood	-8110.00***	-9060.88***	-4703.12***
Sample	14372	14372	6938

Note: * *p* < 0.05; ** *p* < 0.01; *** *p* < 0.001. The reference items are in parentheses.

Table 4 presents the associations between the migration city type and health service use in migration cities, as estimated by logistic regression analysis. Compared with third-tier cities and smaller cities, aged migrant workers who moved to second-tier and first-tier cities were less likely to establish health records and participate in health education than those in western regions; they were also less likely to seek timely medical treatment in the case of illness. This signifies that the more likely that aged migrants are to move to larger cities, the less likely they are to enjoy local public health services.

According to the control variables in Tables 3 and 4, aged migrant workers with health insurance, social security cards, self-assessed health, and hypertension or diabetes were more likely to establish health records and participate in health education programmes. Additionally, the older married migrant workers are more likely to have health records. Younger, male, and better-educated aged migrant workers were more likely to participate in health education programmes. Further, aged migrant workers who had not migrated far, had social security cards, in poor health, and with hypertension or diabetes were also more likely to seek timely medical treatment.

**Table 4:** Associations between migration city type and health services use based on a logistic regression analysis

Variables	Health records	Health education	Medical treatment
***Independent variables***			
Migration city type (third-tier cities and smaller cities)			
Second-tier cities	-0.28(0.04)***	-0.29(0.04)***	-0.24(0.06)***
First-tier cities	-1.12(0.09)***	-0.58(0.06)***	-0.39(0.09)***
***Control variables***			
* Predisposing*			
Age	0.01(0.00)**	-0.02(0.00)***	-0.00(0.00)
Gender (male)			
Female	0.07(0.04)	-0.12(0.04)**	0.01(0.05)
Education (illiterate or primary school)			
Middle school	0.11(0.04)*	0.25(0.04)***	-0.07(0.05)
High school and above	0.03(0.06)	0.31(0.06)***	-0.04(0.08)
Marital status (no spouse)			
Married	0.22(0.07)**	0.05(0.06)	0.03(0.09)
*Enabling*			
Migration range (across counties within a city)			
Across cities, within a province	0.00(0.05)	-0.00(0.05)	-0.19(0.07)**
Across province	-0.20(0.05)***	-0.10(0.05)	-0.10(0.07)
Medical insurance (no)			
Yes	0.24(0.08)**	0.20(0.07)**	0.13(0.10)
Social security card (no)			
Yes	0.48(0.04)***	0.34(0.04)***	0.20(0.05)***
*Needs*			
Self-health assessment (unwell)			
Fair	0.12(0.07)	0.24(0.06)***	-0.35(0.07)***
Health	0.18(0.07)**	0.31(0.06)***	-0.62(0.07)***
Hypertension or diabetes (no)			
Yes	0.25(0.05)***	0.09(0.05)*	0.25(0.06)***
Constants	-2.31(0.24)***	1.15(0.22)***	0.47(0.30)
Log likelihood	-8189.45***	-9214.57***	-4692.86**
Sample	14372	14372	6938

Note: * *p* < 0.05; ** *p* < 0.01; *** *p* < 0.001. The reference items are in parentheses.

Table 5 presents the results for the association between migration destinations and health service use for only healthy aged migrant workers. After eliminating the interference of unhealthy factors, aged migrant workers’ use of health services still revealed regional differences. That is, the results indicated that the better the destination’s economy, the lower the probability of using health services. Healthy aged migrant workers who moved to the eastern regions of China or to first-tier cities were also less likely to access health services.

**Table 5:** Associations between migration city type and health services use among healthy aged migrant workers based on a logistic regression analysis

Variables	Health records	Health education	Medical treatment
**Migration region**			
* Self-assessment is healthy*	*Model 1*	*Model 1*	*Model 1*
Ref: Western	*(n = 8466)*	*(n = 8466)*	*(n = 3266)*
Central	0.42(0.06)***	-0.32(0.06)***	-0.12(0.10)
Eastern	-0.55 (0.06)***	-0.87(0.06)***	-0.19(0.08)*
* no* hypertension or diabetes	*Model 2*	*Model 2*	*Model 2*
Ref: Western	*(n = 11345)*	*(n = 11345)*	*(n = 5085)*
Central	0.45(0.05)***	-0.34(0.05)***	-0.06(0.07)
Eastern	-0.63(0.06)***	-0.91(0.05)***	-0.20(0.07)**
**Migration city type**			
* Self-assessment is healthy*	*Model 3*	*Model 3*	*Model 3*
Ref: third-tier cities and smaller cities	*(n = 8466)*	*(n = 8466)*	*(n = 3266)*
Second-tier cities	-0.18(0.06)**	-0.31(0.05)***	-0.26(0.08)***
First-tier cities	-1.05(0.11)***	-0.57(0.08)***	-0.49(0.12)***
* no* hypertension or diabetes	*Model 4*	*Model 4*	*Model 4*
Ref: third-tier cities	*(n = 11345)*	*(n = 11345)*	*(n = 5085)*
Second-tier cities	-0.25(0.05)***	-0.30(0.05)***	-0.31(0.06)***
First-tier cities	-1.10(0.10)***	-0.59(0.07)***	-0.52(0.11)***

Note: * *p* < 0.05; ** *p* < 0.01; *** *p* < 0.001. Potential confounding variables have been controlled.

## Discussion

Many studies have focused on floating populations’ general nature and related factors or elderly floating populations’ access to public health services in China [3–6]. Less attention has been paid to the health service use among aged migrant workers who live in different types of cities. In this study, the national representative data of China were used to examine the relationship between the migration region and city type of migrant workers aged 50 years and older and their use of health services. The aim of this study was to provide a policy reference for improving the health services use among migrant workers aged 50 years and older.

First, it should be noted that significant regional differences were found regarding aged migrant workers’ use of health services; for instance, aged workers who migrated to the eastern region of China were less likely to use local public health services. In terms of establishing health records, migrants to the central region were more likely to establish health records, while those in the eastern regions had the lowest probability of establishing health records. This finding may have been affected by the relative size of the floating population in the regions. According to the 2016 Report on China’s Migrant Population Development, the floating population in the eastern region in 2015 accounted for 74.7% of the national floating population, while the floating population in the western region accounted for 16.6% [35]. The floating population in the central region was the smallest, while the floating population in in the eastern region was the largest and displayed the greatest rate of mobility. The latter might pose greater challenges to establishing health records for the floating population in the eastern region. Regarding participation in health education, aged migrant workers in the central and eastern regions were more unlikely to participate in health education programmes than those in the western regions—which might strongly correlate with the specific form of health education participation. For example, according to the survey used in this study, the main channels through which aged migrant workers received health education included official publicity material and billboards, and public health consultation activities, which were mainly accessed in residential communities that possessed a larger number of local residents. Both the results of previous studies and those of the current study revealed a residential segregation of migrant workers in urban communities [30]. This was especially true in the eastern regions, where housing costs are relatively high. The relative segregation of residences might hinder the probability of aged migrant workers in the eastern regions receiving health education. In terms of seeking medical treatment, no significant difference was observed in the probability of seeking timely medical treatment for aged workers who migrated to the central and western regions. However, aged workers who migrated to the eastern regions were more likely to delay seeking medical treatment in the case of illness, which may have been influenced by the regional differences in medical costs and the dual urban–rural medical insurance system. Medical costs in the relatively developed eastern regions are higher than those in the central and western regions, but the poor economic conditions of aged migrant workers was a barrier to seeking local medical treatment. Moreover, medical insurance for aged migrant workers was primarily the new rural cooperative medical insurance (NCMS) in the outflow area (their hometowns). When they sought medical treatment away from their hometowns, they faced practical problems such as low reimbursement ratios of medical expenses and complicated reimbursement procedures. This could have lowered the possibility of aged migrant workers seeking timely medical treatment.

The second main point that should be noted for this study is that aged workers who migrated to first- and second-tier cities had a lower probability of using health services than those in third-tier cities and smaller cities. This once again verified the finding that aged migrant workers in more economically developed cities were less likely to access health services. Aged migrant workers in first- or second-tier cities are therefore more likely to be excluded from public health services than those who had migrated to third-tier cities and smaller cities. The reason for this phenomenon should be similar to the one influencing the lower rate of using public health services among the floating populations in the eastern regions. However, compared with migrant workers who moved to a larger cities in the eastern regions, those who moved to first-tier cities, faced higher medical costs, more time to access health services and more severe residential segregation caused by higher housing prices [30, 36]. The influence of these factors reduces the aged migrant workers’ use of health services [28]. For example, China’s first-tier cities are generally characterised by higher housing prices, so aged migrant workers with poor economic conditions tend to live in urban villages and suburbs [29, 30]. The residential segregation of aged migrant workers inevitably affects their health records and health education. Previous studies have also found that floating populations who live in urban areas and in small communities that are dominated by local residents are more likely to establish health records and participate in health education programmes [31, 32]. Additionally, migrant workers’ health consciousness also affects their use of public health services. Studies have demonstrated that migrant workers move to cities for the main purpose of earning money, and that the time and financial costs of accessing medical treatment are relatively high, therefore migrant workers delay seeking medical treatment in case of illness [28]. Other studies have revealed that, compared with urban residents, migrant workers have a strong tolerance for diseases; this can lead to some migrant workers resorting to self-treatment rather than requiring professional medical treatment, which manifests in a low medical consultation rate [36].

This study has offered several meaningful findings regarding the control variables. First, it was found that establishing health records relates to policy orientation. For example, Beijing requires community health service agencies to provide services to key floating population groups—especially children, pregnant women, older people and people with chronic diseases and severe mental disorders, according to China’s Residents Health Records Management System [37]. This study also suggests that aged migrant workers with chronic diseases are more likely to have established health records and that this likelihood increases with age. Second, it was found that the higher the self-assessed health status and educational level of aged migrant workers, the more likely migrant workers will establish health records and participate in health education programmes, which aligns with the findings of previous studies [6]. This might be because people with better education and higher self-assessed health are more likely to understand the benefits of health services. Third, poor self-assessment of health and chronic diseases were found to increase the probability of aged migrant workers seeking timely medical care, which also aligns with the findings of previous studies [4]. This suggests that the actual health demand will increase the probability of aged migrant workers seeking medical care. Fourth, this study found that possessing medical insurance and social security cards can promote aged migrant workers’ use of public health services, which corresponds to the conclusions of previous research [38]. That is, improving the social security system can also improve migrant workers’ use of health services.

Based on the findings of this study, efforts should be dedicated in the following aspects to improve aged migrant workers’ use of public health services. First, attention should be paid to the health services use of aged workers who migrate to the eastern regions and to relatively developed first- and second-tier cities; further, publicity campaigns to encourage migrant workers to establish health records and acquiring health education should be extended to residential areas with large floating populations so that the effect of residential segregation can be reduced. Second, diversified health education approaches should be adopted. In addition to fixed health lectures and health publicity boards, using the internet and mobile health promotion points should also be fully utilised. Third, an urban–rural integrated medical security system should be promoted to lower the threshold of cross-regional medical treatment for aged migrant workers and to simplify procedures, and thus narrow the differences in health services between urban and rural areas and equalise public health services. Last, the floating populations’ health consciousness and health attitudes should be cultivated to change the health behaviours of aged migrant workers.

## Limitations

This study had several limitations. First, the data were horizontal data, which cannot be used to determine causal relationships. Second, the aged migrant workers’ self-expressed use of public health services might lead to deviations in recall. Third, the differences in health service use among aged migrant workers in different migration regions and cities might have been caused by the differences in public service implementation capacity, living and medical costs and other factors in different migration destinations. However, due to the lack of relevant data, this study cannot directly verify the relationship between these factors and aged migrant workers’ use of health services. Fourth, the dependent variables in the study were dichotomous variables, which were used without considering the frequency and intensity of use. Further research and analysis could provide more details about the different effects of migration regions and migration city types.

## Conclusion

This study focuses on the relationship between migration regions and city types and aged migrant workers’ use of public health services in China. Inequality was observed in the public services of different cities, and public health services use by aged migrant workers was not consistent with the city economy. Aged workers who had migrated to relatively developed eastern regions and first- and second-tier cities were also less likely to use public health services. This may be attributed to the relatively high living costs and residential isolation in developed regions and cities that limit aged migrant workers’ access to public health services. To achieve health equality, more attention must be paid to public health services for aged workers who migrate to developed regions and cities.

## Data Availability

Since the data used in this paper were provided by China’s National Population and Family Planning Commission, which is the top agency governing health issues in China, we had to sign a legally binding agreement with the Commission that we will not share any original data with any third party. However, interested researchers can apply for access to the data at http://www.chinaldrk.org.cn/wjw/#/application/index and e-mail:ldrkzxsj@163.com.
